# Molecular modulation of cartilage graft survival: a systematic review and translational synthesis of local and systemic strategies

**DOI:** 10.1016/j.jpra.2026.08.001

**Published:** 2026-08-10

**Authors:** Mehmet Sefik Oruc, Ali Can Gunenc, Furkan Yılmaz, Ovunc Akdemir

**Affiliations:** Department of Plastic, Reconstructive and Aesthetic Surgery, Faculty of Medicine, Istanbul Aydın University, Istanbul, Türkiye

**Keywords:** Cartilage graft, Graft survival, Platelet-rich plasma, Platelet-rich fibrin, Perichondrium, Regenerative medicine

## Abstract

**Background:**

Cartilage grafting is widely used in reconstructive and aesthetic surgery, but graft resorption, volume loss, reduced chondrocyte viability, and variable graft-host integration remain important limitations. This review evaluated preclinical evidence for technical graft-preparation strategies and local or systemic biologic approaches intended to improve cartilage graft survival.

**Methods:**

A systematic review of preclinical in vivo evidence was reported in accordance with PRISMA 2020. PubMed/MEDLINE, Scopus, Web of Science, and Google Scholar were searched between April 1 and May 1, 2026, with a publication cutoff of March 31, 2026. Direct graft-specific comparative studies, including technical preparation/interface strategies and biologically active interventions, were distinguished from broader contextual publications. Because of heterogeneity in models, strategies, follow-up, and outcomes, meta-analysis and inferential comparisons were not performed. Findings were synthesized narratively, and risk of bias in direct animal studies was assessed using SYRCLE's tool.

**Results:**

Thirty-three publications informed the review: 14 direct graft-specific studies, 5 technical or clinical contextual publications, and 14 indirect mechanistic publications. Direct evidence was predominantly derived from rabbit models and included technical graft-preparation/interface comparisons and locally delivered biologic interventions such as platelet-derived products, fibrin-based materials, and perichondrial support. Several studies reported favorable graft-related findings, but benefits could not be compared reliably across strategy categories. No direct graft-specific systemic intervention study was identified; the systemic rationale was supported by indirect literature on oxidative stress, inflammation, apoptosis, extracellular matrix biology, and tissue repair. Methodological reporting was variable, with several risk-of-bias domains remaining unclear.

**Conclusion:**

Several technical graft-preparation approaches and local biologic strategies have shown promising findings in preclinical cartilage graft models. However, substantial heterogeneity and methodological limitations preclude conclusions regarding comparative superiority or routine clinical efficacy. Systemic and combined strategies remain investigational, and the proposed framework is hypothesis-generating pending standardized preclinical studies and prospective clinical validation.

## Introduction

Cartilage grafting is a cornerstone technique in reconstructive and aesthetic surgery because of its structural stability, low immunogenicity, and long-term versatility.^1^^,^^2^ It is widely used in rhinoplasty, auricular reconstruction, and craniofacial procedures; however, unpredictable resorption, loss of volume, contour irregularity, and reduced chondrocyte viability may compromise long-term outcomes.^3^, ^4^, ^5^, ^6^

Experimental and clinical observations indicate that graft behavior is influenced by graft preparation, mechanical manipulation, recipient-site characteristics, and the surrounding biological microenvironment.^3^^,^^7^^,^^8^ Diced, crushed, block, fascia-wrapped, and scaffold-assisted grafts demonstrate different patterns of viability, resorption, incorporation, and structural preservation.^2^^,^^4^^,^^9^^,^^10^ Technical optimization therefore remains important, but it does not fully explain the variability of long-term graft performance.^19^^,^^20^

Interest has consequently shifted toward biologic modulation of the graft-recipient interface. Local strategies, including platelet-rich plasma (PRP), platelet-rich fibrin (PRF), concentrated growth factor (CGF), autologous fibrin, and perichondrial support, have been investigated as methods of preserving chondrocyte viability, extracellular matrix integrity, and graft incorporation.^11^, ^12^, ^13^, ^14^, ^15^, ^16^, ^17^, ^18^

Systemic or host-environment modulation is biologically plausible but supported by a less direct evidence base. Publications concerning antioxidant activity, inflammation, apoptosis, extracellular matrix metabolism, angiogenesis, and tissue repair suggest pathways that may influence graft fate, but most do not directly evaluate survival of an implanted cartilage graft.^21^, ^22^, ^23^, ^24^, ^25^, ^26^, ^27^, ^28^, ^29^, ^30^, ^31^, ^32^, ^33^ The distinction between direct graft-specific evidence and indirect mechanistic support is therefore essential.

The present study was designed as a systematic review of preclinical in vivo cartilage graft evidence, followed by a structured narrative and translational synthesis. Its objectives were to summarize direct experimental findings, distinguish them from indirect mechanistic literature, assess methodological risk of bias, and propose a clearly labeled hypothesis-generating framework for future research rather than a clinical treatment hierarchy.

## Methods

### Study design and reporting framework

This study was conducted as a systematic review of preclinical in vivo evidence, followed by a structured narrative and translational synthesis. The identification, screening, eligibility assessment, and inclusion of graft-specific experimental studies were reported in accordance with PRISMA 2020.^34^

The systematic review component comprised the predefined literature search, record management, eligibility assessment, study selection, data extraction, and appraisal of direct graft-specific studies. The term translational synthesis refers only to the subsequent organization and interpretation of the evidence across biologically relevant domains and its potential relevance to plastic, reconstructive, and aesthetic surgery. It was not used as an alternative study-selection methodology.

### Review question and scope

The primary review question was whether technical graft-preparation or interface strategies, or local, systemic, or combined molecular and biologically active interventions, influence the preservation, viability, resorption, integration, or structural durability of implanted cartilage grafts in preclinical in vivo models.

Studies involving auricular, septal, costal, or other structurally implanted cartilage grafts were considered potentially eligible. Composite grafts, vascularized grafts, and tissue-engineered constructs without an interpretable cartilage graft survival endpoint were excluded from the direct evidence synthesis. Broader articular cartilage and tissue-repair publications were retained only when they provided mechanistic context relevant to chondrocyte viability, extracellular matrix preservation, oxidative stress, inflammation, apoptosis, angiogenesis, or graft-host interaction.

### Evidence classification

Publications were organized into three evidence categories. Direct graft-specific evidence comprised preclinical in vivo comparative studies evaluating a structurally implanted cartilage graft under a technical graft-preparation or interface strategy, or a local, systemic, or combined biologically active intervention, with at least one interpretable graft-related outcome. These studies formed the primary evidence base and were included in the descriptive synthesis and risk-of-bias assessment.

Technical or clinical contextual publications described graft behavior, preparation, incorporation, or clinical use but were not treated as preclinical intervention-effect evidence. Indirect mechanistic evidence addressed biological processes potentially relevant to graft survival without directly evaluating an implanted cartilage graft intervention. Both contextual categories were used only for interpretation and were excluded from outcome-direction comparisons and risk-of-bias assessment.

### Data sources and search strategy

Searches were conducted sequentially in PubMed/MEDLINE between April 1 and April 4, 2026; Scopus between April 5 and April 8, 2026; Web of Science between April 10 and April 15, 2026; and Google Scholar between April 17 and May 1, 2026. The final search was completed on May 1, 2026. To ensure a consistent temporal scope across all sources, records published from database inception through March 31, 2026, were eligible.

The search combined cartilage graft-related terms with terms describing platelet-derived products, fibrin-based materials, perichondrial support, scaffolds, antioxidants, anti-inflammatory agents, growth factors, and other biologically active interventions. Controlled vocabulary and free-text terms were used where available. PubMed/MEDLINE searches combined Medical Subject Headings with title and abstract terms; equivalent concepts were translated to title, abstract, and keyword fields in Scopus and the Topic field in Web of Science. Google Scholar was searched using shorter predefined combinations because of its less structured search interface. Complete database-specific strategies, field codes, limits, and search dates are provided in Supplementary Table S1.

Reference lists of eligible graft-specific studies and relevant reviews were screened manually to identify additional potentially eligible publications. Only English-language publications were included.

The searches yielded 536 records from PubMed/MEDLINE, 318 from Scopus, 406 from Web of Science, and 430 records screened and exported from Google Scholar, for a total of 1690 records before deduplication. Manual reference screening identified no additional unique records.

### Eligibility criteria

Studies were eligible for the direct evidence synthesis when they used an in vivo implanted cartilage graft model; evaluated a technical graft-preparation or interface strategy, or at least one local, systemic, or combined biologically active intervention; reported at least one interpretable graft-related outcome; and provided sufficient methodological information to determine the strategy, route or interface, timing, and outcome direction.

Eligible graft-related outcomes included gross graft preservation, volume or weight retention, resorption, histological integrity, chondrocyte viability, extracellular matrix preservation, inflammatory response, graft-host integration, and biomechanical or structural durability.

Studies were excluded from the direct evidence synthesis if they were in vitro only; were clinical case reports without an experimental design; evaluated composite or vascularized grafts without separable cartilage-specific findings; assessed tissue-engineered constructs without direct graft-survival outcomes; or provided insufficient methodological or outcome information. Publications that did not meet direct-evidence eligibility but contributed relevant technical, clinical, or mechanistic context were classified separately and were not used to infer intervention efficacy.

### Study selection and independent verification

All records retrieved from PubMed/MEDLINE, Scopus, Web of Science, and Google Scholar were imported into EndNote and combined in a single reference library. Duplicate records were identified using the EndNote duplicate-detection function and were manually reviewed before removal.

The initial title and abstract screening was performed by MSO. During revision, OA independently reassessed all 80 full-text reports, confirmed the inclusion of 33 publications and the exclusion decisions and reasons for 47 reports, and independently verified evidence classification, extracted data, and risk-of-bias judgments.

Reasons for full-text exclusion were documented using predefined categories. Each excluded report was assigned to a single primary exclusion category to avoid double counting. The categories and frequencies are summarized in Supplementary Table S4. Disagreements regarding eligibility, evidence classification, exclusion reasons, extracted data, or risk-of-bias judgments were resolved through discussion and consensus, with ACG serving as the third reviewer when required.

### Data extraction and mechanistic mapping

For each direct graft-specific study, the following variables were extracted: animal species and model, cartilage source, graft preparation, implantation site, technical strategy or intervention type, administration route when applicable, timing and duration of treatment, follow-up interval, comparator, gross findings, histological outcomes, viability findings, matrix-related outcomes, integration findings, and mechanistic markers.

Direct findings were mapped to predefined biological domains: oxidative stress regulation, inflammatory signaling, chondrocyte viability and apoptosis, extracellular matrix preservation, graft-host integration, and structural durability. This mapping was used to organize heterogeneous findings and did not establish causality or confirm that an intervention acted exclusively through a single pathway.

### Risk-of-bias assessment

The methodological risk of bias of direct in vivo comparative animal studies was assessed using SYRCLE's Risk of Bias Tool for Animal Studies.^35^ The tool evaluates sequence generation, baseline characteristics, allocation concealment, random housing, blinding of caregivers and investigators, random outcome assessment, blinding of outcome assessors, incomplete outcome data, selective outcome reporting, and other potential sources of bias.

Each domain was independently rated as low risk, high risk, or unclear risk by MSO and OA. An unclear judgment was assigned when the report did not provide sufficient information to support a low- or high-risk judgment. Disagreements were resolved by consensus, with unresolved judgments referred to ACG. No study was excluded solely because of its risk-of-bias judgment; the appraisal was used to inform the interpretation of the narrative synthesis. Domain-level judgments are presented in Supplementary Table S2.

### Narrative synthesis and descriptive outcome assessment

Quantitative meta-analysis was not performed because the included studies differed substantially in animal species, cartilage source, graft dimensions, implantation site, intervention type and dose, treatment duration, follow-up interval, outcome definition, and experimental unit. In addition, numerical effect estimates and variance measures were not reported consistently, and some studies evaluated multiple grafts within the same animal.

Direct graft-specific studies were grouped by strategy type, intervention route, and intervention class. For outcome domains explicitly evaluated in an individual study, findings were categorized descriptively as favorable, mixed, neutral, unfavorable, or not assessed. Outcomes that were not measured or insufficiently reported were treated as not assessed and were excluded from the corresponding denominator.

Each study contributed only once to an individual outcome domain. Because a single study could contribute to several correlated domains, cross-domain observations were not treated as statistically independent. No chi-square test, Fisher's exact test, pooled cross-domain percentage, or other inferential comparison was performed between local and systemic intervention categories. The study-level descriptive outcome map is provided in Supplementary Table S3.

### Translational interpretation

After completion of the direct evidence synthesis, findings were considered in relation to clinically relevant problems such as graft resorption, contour loss, weakening of structural support, and impaired graft-host integration. These interpretations were used to formulate research hypotheses and were not treated as clinical recommendations.

The author-proposed framework integrates direct graft-specific observations with indirect mechanistic literature. It is explicitly conceptual and hypothesis-generating and should not be interpreted as a validated causal model, comparative treatment hierarchy, or clinical algorithm.

### Ethics and generative AI statements

This review analyzed previously published literature and involved no new human participants or experimental animals; additional ethical approval was therefore not required.

Artificial intelligence-assisted tools were used solely for language refinement and graphical rendering support. All scientific decisions, study classifications, risk-of-bias judgments, interpretations, and final editorial decisions were made and verified by the authors.

## Results

### Study selection and evidence profile

**Fig. 2:**
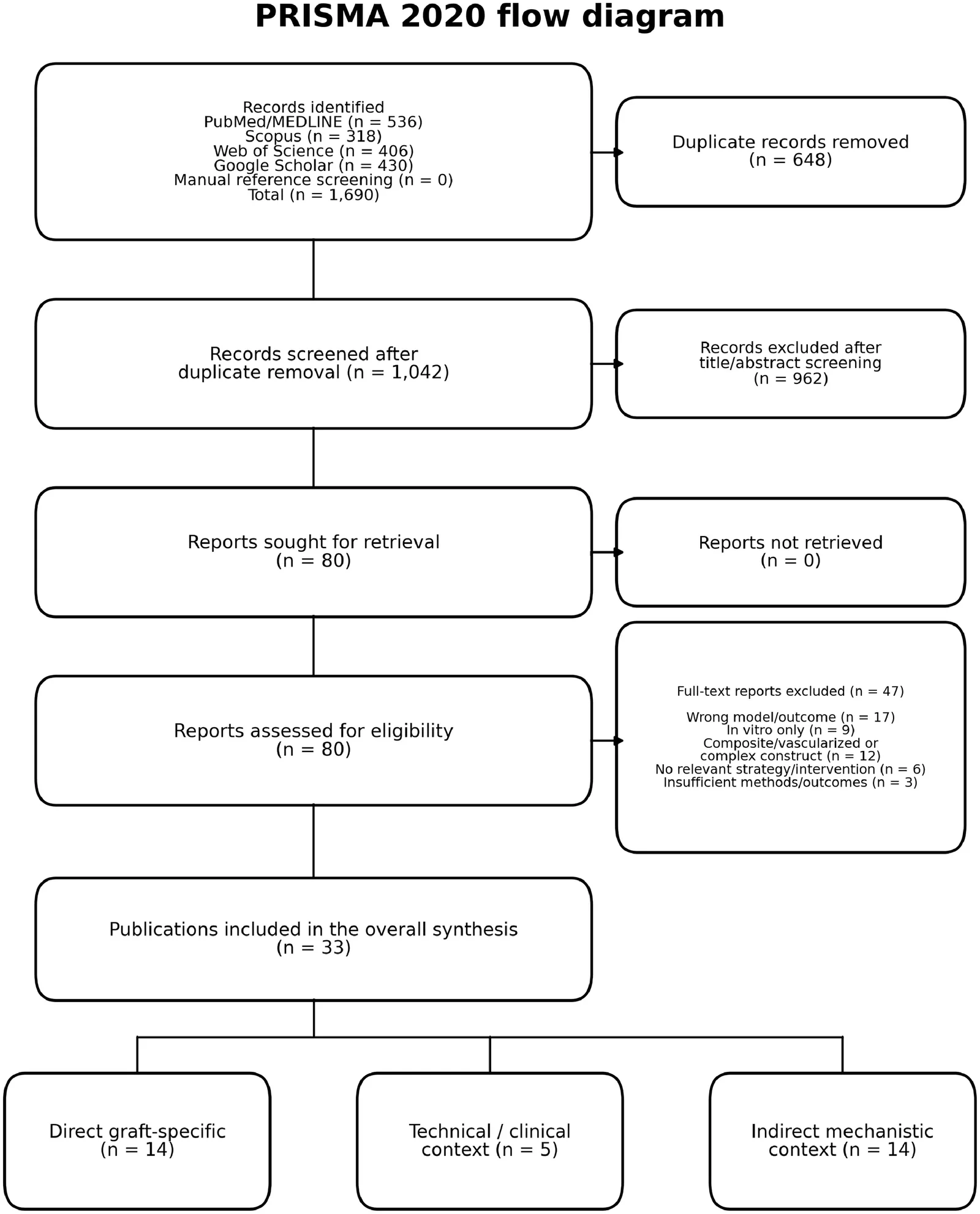
PRISMA 2020 flow diagram of study identification, screening, eligibility, and inclusion. It presents database counts, manual screening, duplicate removal, title and abstract screening, full-text assessment, exclusion categories, and final evidence classification. Definitions are provided in Supplementary Table S4. Graphical layout was prepared with OpenAI ChatGPT assistance; all counts and labels were verified by the authors.

The searches identified 1690 records: 536 from PubMed/MEDLINE, 318 from Scopus, 406 from Web of Science, and 430 from Google Scholar. Manual reference screening identified no additional unique records. After removal of 648 duplicates, 1042 records underwent title and abstract screening; 962 were excluded. Eighty reports were retrieved and assessed in full text. Forty-seven reports were excluded for the following primary reasons: wrong experimental model or outcome (n = 17), in vitro-only design (n = 9), composite, vascularized, or complex constructs without a separable cartilage graft endpoint (n = 12), no relevant technical or biologic strategy (n = 6), and insufficient methodological or outcome information (n = 3). The remaining 33 publications informed the review: 14 direct graft-specific studies, 5 technical or clinical contextual publications, and 14 indirect mechanistic publications. Detailed definitions of the full-text exclusion categories are provided in Supplementary Table S4, and the selection process is summarized in Fig. 2.

The direct graft-specific evidence base was predominantly preclinical and animal based, with rabbit models representing the most frequently used platform. Technical graft-preparation/interface studies and locally delivered biologic interventions together constituted the direct evidence base. Technical or clinical publications provided contextual background, whereas indirect mechanistic publications were used solely to support biological plausibility. Characteristics of the 14 direct graft-specific studies are summarized in Table 1. A concise synthesis of the evidence by intervention category is provided in Table 2.

**Table 1:** Direct graft-specific in vivo studies: design and principal finding.

Study	Model / intervention	Principal finding
Cakmak et al., 20,05^3^	Rabbit; intact, diced, and graded crushing	Viability decreased as crushing severity increased.
Kim et al., 20,11^4^	Rabbit; bare, fascia-, or AlloDerm-wrapped diced cartilage	AlloDerm improved selected histologic findings only.
Fatemi et al., 20,12^5^	Rabbit; block versus fascia-wrapped diced cartilage	Block grafts showed better structural preservation.
Firat et al., 20,11^6^	Rabbit; graft form, wrapping, and preservation methods	Bare grafts showed the highest viability.
Kayabasoglu et al., 20,15^7^	Rabbit; diced, crushed, and morselized cartilage	Diced cartilage retained more viable cells.
Kemaloglu et al., 20,14^8^	Rabbit; perichondrium versus fascia wrapping	Perichondrium improved chondrocyte viability.
Brenner et al., 200,6^10^	Nude rat; free, Surgicel-, or fascia-wrapped diced cartilage	Fascia preserved more viable cartilage than Surgicel.
Manafi et al., 201,2^11^	Rabbit; local PRP versus saline	PRP improved regenerative and angiogenic findings.
Göral et al., 201,6^12^	Rabbit; PRF versus wrapping controls	PRF improved viability relative to oxidized cellulose.
Topkara et al., 201,6^13^	Rabbit; CGF and wrapping comparators	CGF produced the most favorable proliferation findings.
Aksan et al., 202,1^14^	Rabbit; autologous fibrin-adhered diced cartilage	Fibrin increased proliferation; other outcomes were similar.
Kulaksiz et al., 202,4^15^	Rabbit; PRP or CGF versus control	Both products improved injury and regeneration scores.
Shi et al., 202,0^16^	Rabbit; perichondrium preserved versus removed	Preservation improved viability and biomechanical properties.
Hapsari et al., 202,2^17^	Rabbit; perichondrial attachment/wrapping strategies	Attachment increased proliferation and reduced apoptosis.

Findings are summarized at study level and are not pooled treatment effects. Detailed risk-of-bias judgments are provided in Supplementary Table S2.

**Table 2:** Concise evidence synthesis by intervention category.

Evidence category	Direct evidence	Concise synthesis
Mechanical preparation and graft form^3^^,^^5^, ^6^, ^7^	4 studies	Less traumatic handling generally preserved viability; findings remained model dependent.
Wrapping and interface materials^4^^,^^10^	2 studies	Effects varied by material and experimental model.
Platelet-derived products^11^, ^12^, ^13^^,^^15^	4 studies	Several studies reported favorable viability or regeneration findings; comparative efficacy is unproven.
Autologous fibrin^14^	1 study	A favorable proliferation finding requires independent replication.
Perichondrial support^8^^,^^16^^,^^17^	3 studies	Cellular preservation was favorable, but clinical relevance remains uncertain.
Systemic or host-environment modulation^21^^–^^33^	No verified direct study	Mechanistic rationale is indirect and does not establish treatment efficacy.

Reference 18 and references 21–33 provide indirect mechanistic context and were not treated as direct intervention-efficacy evidence.

### Risk-of-bias assessment

Domain-level risk-of-bias judgments are presented in Supplementary Table S2. Methodological reporting varied across studies, and several judgments remained unclear because randomization, allocation concealment, random housing, and blinding procedures were incompletely described. These limitations were considered when interpreting the consistency and translational relevance of reported intervention effects.

### Direct graft-specific evidence: technical preparation and interface strategies

Technical comparative studies evaluated graft form, degree of crushing or morselization, and wrapping or interface materials.^3^, ^4^, ^5^, ^6^, ^7^, ^8^, ^9^, ^10^ Less traumatic preparation generally preserved viability, while the effects of fascia, AlloDerm, oxidized cellulose, and perichondrial wrapping varied across models. These findings were study-specific and did not establish a universally superior preparation method.

### Direct graft-specific evidence: local interventions

Local biologic and biomaterial strategies included PRP, PRF, CGF, autologous fibrin, and perichondrial support.^8^^,^^11^, ^12^, ^13^, ^14^, ^15^, ^16^, ^17^ Several studies reported favorable findings in one or more domains, including gross preservation, reduced resorption, histological architecture, viable chondrocyte counts, extracellular matrix preservation, or graft integration.

Platelet-derived products were associated with favorable findings in multiple experimental models.^11^, ^12^, ^13^^,^^15^ Perichondrium-containing strategies were also associated with preservation of cellular or matrix-related outcomes in selected studies.^8^^,^^16^^,^^17^ However, the protocols, comparators, graft forms, follow-up intervals, and outcome measures differed substantially, and the available evidence did not permit quantitative estimation of a common treatment effect.

### Systemic and combined strategies

No direct graft-specific study of a systemically administered intervention was identified. Accordingly, a comparative conclusion between systemic and local routes could not be drawn.

Combined or multi-component approaches were summarized separately when the independent contribution of individual components could not be determined. These findings were treated as exploratory and were not used to infer additive or synergistic efficacy.

### Indirect mechanistic evidence

Broader publications concerning perichondrial biology, oxidative stress, inflammation, apoptosis, extracellular matrix metabolism, angiogenesis, and tissue repair provided biological context for potential determinants of cartilage graft fate.^18^^,^^21^, ^22^, ^23^, ^24^, ^25^, ^26^, ^27^, ^28^, ^29^, ^30^, ^31^, ^32^, ^33^ These publications did not directly demonstrate improved survival of implanted cartilage grafts and were therefore not included in the direct intervention-effect synthesis.

**Fig. 1:**
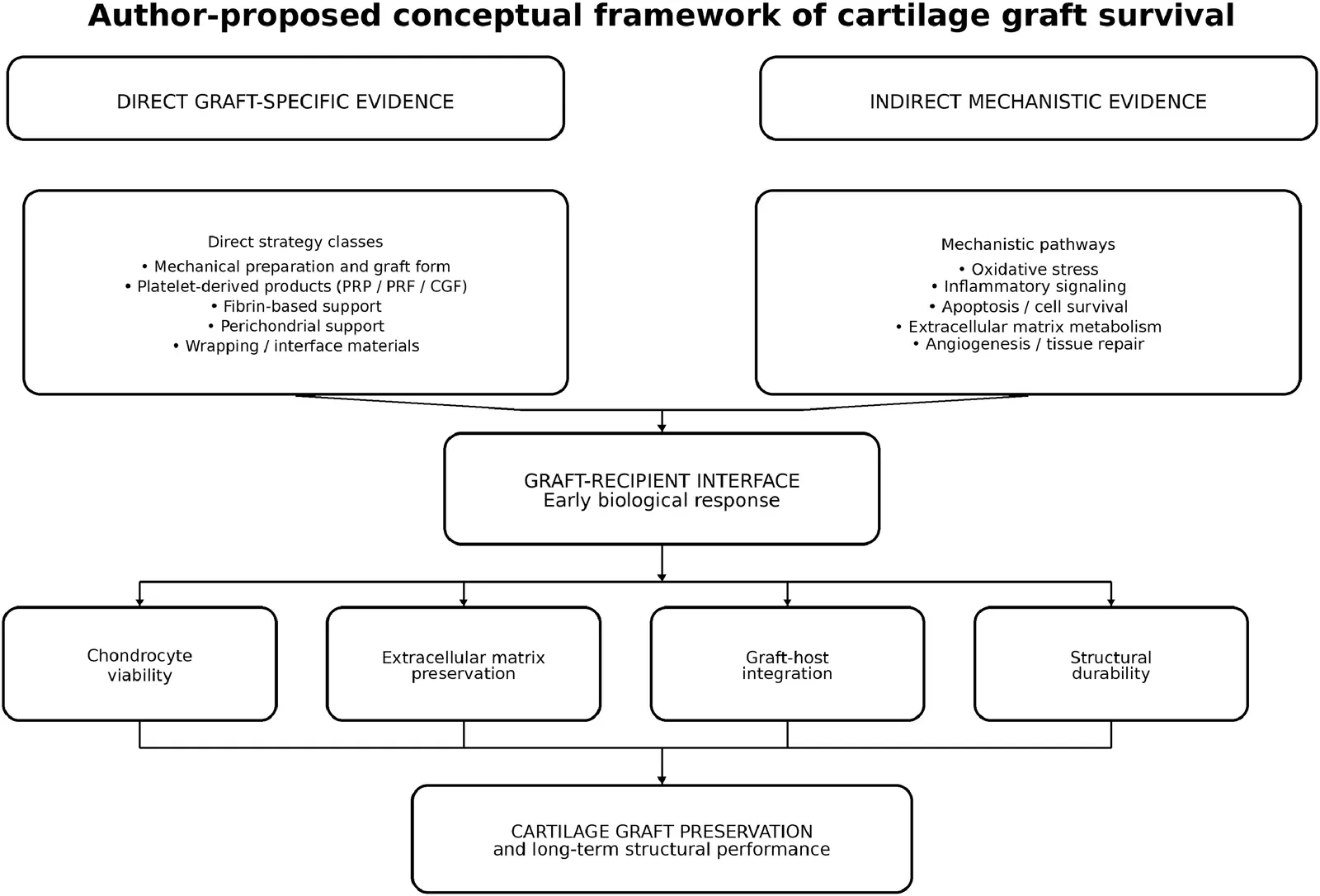
Author-proposed conceptual framework of factors influencing cartilage graft survival. Direct strategies include graft preparation, interface approaches, and local biologic interventions; systemic or host-environment pathways provide indirect mechanistic support. The framework links oxidative stress, inflammation, chondrocyte viability, extracellular matrix preservation, graft-host integration, and structural durability across the graft-recipient interface. It is hypothesis-generating and not a validated causal model, treatment hierarchy, or clinical recommendation. Graphical layout was prepared with OpenAI ChatGPT assistance and finalized by the authors.

Collectively, the indirect literature supports the plausibility that oxidative injury, excessive inflammatory signaling, loss of chondrocyte viability, matrix degradation, and impaired graft-host interaction may influence graft preservation. These mechanisms informed the author-proposed conceptual framework shown in Fig. 1.

### Descriptive outcome synthesis

Study-level outcome direction was summarized only for domains explicitly evaluated in each direct graft-specific publication. Favorable, mixed, neutral, unfavorable, and not-assessed classifications are presented in Supplementary Table S3.

Several technical-strategy and local-intervention studies reported favorable findings, but the number of studies contributing to each outcome domain differed and the underlying models were not directly comparable. Because individual studies frequently contributed to multiple correlated domains, no pooled cross-domain proportions or inferential statistical comparisons were calculated. The descriptive synthesis should therefore be interpreted as an evidence map rather than proof of comparative efficacy.

### Translational interpretation

The direct evidence supports continued investigation of technical graft preparation and graft-interface augmentation, while the indirect literature provides a rationale for studying host-environment modulation. The relationship between direct strategies, systemic biological pathways, and potential determinants of graft fate is summarized in the author-proposed framework in Fig. 1.

This framework does not establish treatment superiority or justify routine clinical use. Rather, it identifies testable pathways and comparative study designs for future preclinical and clinical investigation.

## Discussion

### Principal findings

This review identified a preclinical evidence base comprising technical graft-preparation/interface comparisons and locally delivered biologic strategies. Less traumatic preparation, selected wrapping or perichondrial approaches, platelet-derived products, and fibrin-based materials were associated with favorable findings in several individual cartilage graft models.^3^, ^4^, ^5^, ^6^, ^7^, ^8^, ^9^, ^10^, ^11^, ^12^, ^13^, ^14^, ^15^, ^16^, ^17^

These findings are promising but should not be interpreted as proof that local interventions are superior to systemic strategies. The evidence bases differed substantially in size, intervention type, model design, outcome selection, follow-up, and methodological quality. Removal of the previous pooled domain-level comparison avoids attributing statistical significance to correlated outcomes derived from the same studies.

### Direct evidence and indirect mechanistic context

A central revision of this review is the explicit separation of direct graft-specific evidence from contextual publications. Direct evidence is restricted to in vivo implanted cartilage graft studies evaluating a technical preparation/interface strategy or biologically active intervention with interpretable graft-related outcomes. Technical or clinical contextual publications describe graft use or behavior without contributing preclinical intervention-effect estimates, while broader mechanistic literature is used only to explain biological plausibility.^18^, ^19^, ^20^, ^21^, ^22^, ^23^, ^24^, ^25^, ^26^, ^27^, ^28^, ^29^, ^30^, ^31^, ^32^, ^33^

This distinction is particularly important for systemic modulation. Although systemic antioxidant or anti-inflammatory approaches are biologically plausible, no direct graft-specific systemic intervention study was identified. Mechanistic plausibility should therefore not be presented as evidence of clinical or graft-specific efficacy.

### Cartilage graft survival as a multi-domain biological process

Traditional surgical considerations such as harvesting technique, crushing force, diced particle size, wrapping material, and recipient-site preparation remain important determinants of graft behavior.^2^, ^3^, ^4^, ^5^, ^6^, ^7^, ^8^, ^9^, ^10^^,^^19^^,^^20^ The available literature also suggests that graft fate may be influenced by interacting biological domains involving oxidative stress, inflammatory signaling, chondrocyte viability, extracellular matrix integrity, and graft-host integration.

Reactive oxygen species may impair cell metabolism and accelerate matrix degradation.^24^^,^^25^ Excessive inflammatory signaling may promote catabolic remodeling.^23^^,^^24^ Chondrocyte survival is relevant to matrix maintenance,^28^, ^29^, ^30^, ^31^, ^32^, ^33^ while interface healing and surrounding tissue support may affect incorporation and structural persistence.^26^^,^^27^ These relationships are biologically plausible but should be understood as an interpretative synthesis rather than a validated causal sequence.

### Potential explanations for favorable findings with local interventions

One possible explanation for favorable findings in local-intervention studies is pharmacobiological proximity. Locally applied agents are delivered directly to the graft-recipient interface, where early cellular survival, diffusion, matrix remodeling, and interface healing occur. This may be relevant because cartilage is relatively avascular.

Nevertheless, this explanation remains hypothesis-generating. Differences between local and systemic evidence bases preclude a valid route-to-route efficacy comparison. The current literature supports further investigation of local delivery approaches but does not establish their preferential or routine clinical use.

### Combined and multi-target strategies

Cartilage graft degeneration may involve several interacting pathways rather than a single dominant mechanism. Combined local and systemic strategies may therefore warrant investigation, particularly when they target complementary biological processes.

However, additive or synergistic efficacy has not been established. Future head-to-head studies should compare local therapy alone, systemic therapy alone, combined therapy, and appropriate controls using standardized graft models and predefined outcomes.

### Clinical relevance and research implications

The available evidence is insufficient to support a validated clinical algorithm or routine biologic augmentation of cartilage grafts. The principal clinical relevance of the present synthesis is the identification of testable research hypotheses.

In rhinoplasty, auricular reconstruction, craniofacial contour restoration, and revision surgery, future studies could evaluate whether biologic augmentation improves volume retention, viable chondrocyte density, mechanical support, or graft-host integration. Such studies should first establish reproducible efficacy and safety in standardized preclinical models before prospective clinical translation.

### Author-proposed translational framework

Integrating direct graft-specific findings with indirect mechanistic literature, the authors propose a framework in which local strategies primarily influence the graft-recipient interface, whereas systemic strategies may theoretically modify the broader host environment through oxidative, inflammatory, or regenerative pathways.

This framework is conceptual and hypothesis-generating. It does not represent a validated causal model, comparative treatment hierarchy, or clinical recommendation. Its purpose is to organize future experimental questions and clarify which assumptions require direct testing.

### Limitations

This review has several limitations. First, the included studies were heterogeneous in species, cartilage source, graft form, graft size, implantation site, intervention protocol, follow-up, and outcome definition, preventing quantitative meta-analysis.

Second, many studies emphasized histological findings, while validated viability assays, volumetric measurements, and long-term biomechanical outcomes were reported less consistently. Outcome-direction synthesis does not capture effect magnitude, precision, or sample size and should be interpreted as a descriptive evidence map.

Third, methodological reporting was incomplete in several studies, producing unclear risk-of-bias judgments. Animal models cannot fully reproduce the mechanical loading, scar biology, recipient-site variability, and long-term clinical conditions encountered in human reconstruction.

Fourth, the original screening was undertaken by one reviewer. Independent reassessment during revision reduced, but cannot entirely eliminate, the possibility of selection or classification bias inherent in a retrospectively verified process.

Fifth, the review was not prospectively registered, and publication bias remains possible because favorable experimental findings may be more likely to be published. Finally, mechanistically supportive publications were intentionally included for contextual interpretation, but they should not be regarded as direct evidence of graft benefit.

### Future directions

Future research should prioritize standardized animal models, reproducible graft dimensions and implantation sites, transparent randomization and blinding, prespecified outcome definitions, and reporting of numerical effect estimates with variance measures.

Direct comparative studies of PRP, PRF, CGF, fibrin-based supports, and perichondrial strategies are needed. Studies should also evaluate dose-response relationships, optimal timing, long-term structural durability, and whether combined local and systemic approaches provide benefit beyond local treatment alone.

Prospective clinical studies should be considered only after reproducible preclinical efficacy and safety have been established. Molecular profiling of successful and failed grafts may help identify new therapeutic targets and improve biological stratification.

## Conclusion

Current preclinical evidence indicates that several technical graft-preparation/interface strategies and local biologic interventions, particularly less traumatic handling, selected wrapping or perichondrial approaches, platelet-derived products, and fibrin-based materials, have been associated with favorable cartilage graft-related findings in experimental models.

However, substantial heterogeneity in animal models, graft configurations, intervention protocols, follow-up intervals, outcome definitions, and methodological quality prevents conclusions regarding comparative superiority. No direct graft-specific systemic intervention study was identified, and the systemic rationale is derived from indirect mechanistic literature.

Technical, local biologic, systemic, and combined strategies should therefore be regarded as investigational. The proposed biological framework is hypothesis-generating and requires validation through standardized preclinical studies and prospective clinical research before evidence-based recommendations can be established.

## Author contributions

MSO: Conceptualization, methodology, literature search, initial title and abstract screening, data extraction, synthesis, visualization, and writing - original draft. ACG: Methodological supervision, adjudication of unresolved eligibility and risk-of-bias judgments, interpretation, and writing - review and editing. FY: Data organization, literature verification, interpretation, and writing - review and editing. OA: Independent full-text eligibility reassessment, evidence classification and data verification, risk-of-bias assessment, supervision, and writing - review and editing. All authors reviewed and approved the final manuscript.

## Funding

The authors received no financial support for the research, authorship, and/or publication of this article.

## Data availability statement

All data analyzed in this review were derived from previously published articles cited in the manuscript. No new primary dataset or analytic code was generated. The database-specific search strategies, full-text exclusion summary, risk-of-bias judgments, and study-level outcome-direction data are provided in Supplementary Tables S1-S4.

## Ethics statement

This review analyzed previously published studies and involved no new human participants or experimental animals; additional ethical approval was not required.

## Declaration of generative AI statement

During preparation of this work, the authors used OpenAI ChatGPT for language refinement, formatting, consistency checking, and graphical support. All AI-assisted content was reviewed and verified by the authors, who take full responsibility for the manuscript.

## Declaration of competing interest

The authors declare that there are no conflicts of interest related to this work.

## Appendix Supplementary materials

Image, application 1

Image, application 2

Image, application 3

Image, application 4
