# Supplementary material for: Molecular modulation of cartilage graft survival: a systematic review and translational synthesis of local and systemic strategies
Source: JPRAS Open. 2026 Aug 10;52:128–37. doi: 10.1016/j.jpra.2026.08.001 (PMC13560031; doi:10.1016/j.jpra.2026.08.001)
Supplement: Supplementary file 1 [file mmc1.docx]

**Supplementary Table S1. Database-specific search strategies**

**PubMed/MEDLINE**

**Search date:** April 1-4, 2026

**Direct graft-specific search**

(("Cartilage/transplantation"[Mesh] OR "cartilage graft"[Title/Abstract] OR "cartilage grafts"[Title/Abstract] OR "cartilage graft survival"[Title/Abstract] OR "cartilage graft resorption"[Title/Abstract] OR "cartilage transplantation"[Title/Abstract] OR "cartilage implantation"[Title/Abstract] OR "diced cartilage"[Title/Abstract] OR "crushed cartilage"[Title/Abstract] OR "morselized cartilage"[Title/Abstract] OR "morselised cartilage"[Title/Abstract]) AND ("graft preparation"[Title/Abstract] OR "cartilage preparation"[Title/Abstract] OR crushing[Title/Abstract] OR dicing[Title/Abstract] OR morselization[Title/Abstract] OR morselisation[Title/Abstract] OR wrapping[Title/Abstract] OR fascia[Title/Abstract] OR AlloDerm[Title/Abstract] OR Surgicel[Title/Abstract] OR "Platelet-Rich Plasma"[Mesh] OR "platelet-rich plasma"[Title/Abstract] OR "platelet-rich fibrin"[Title/Abstract] OR "concentrated growth factor"[Title/Abstract] OR "fibrin matrix"[Title/Abstract] OR "autologous blood fibrin"[Title/Abstract] OR perichondri*[Title/Abstract] OR scaffold*[Title/Abstract] OR biomaterial*[Title/Abstract] OR antioxidant*[Title/Abstract] OR "anti-inflammatory"[Title/Abstract] OR melatonin[Title/Abstract] OR "vitamin C"[Title/Abstract] OR "ascorbic acid"[Title/Abstract] OR glutathione[Title/Abstract] OR "growth factor"[Title/Abstract] OR "growth factors"[Title/Abstract] OR "biologic augmentation"[Title/Abstract] OR "biological augmentation"[Title/Abstract])) AND English[Language]

**Contextual mechanistic search**

("cartilage"[Title/Abstract] OR chondrocyte*[Title/Abstract]) AND ("oxidative stress"[Title/Abstract] OR inflammation[Title/Abstract] OR apoptosis[Title/Abstract] OR "extracellular matrix"[Title/Abstract] OR angiogenesis[Title/Abstract] OR "tissue repair"[Title/Abstract]) AND English[Language]

**Limits and coverage:** English language; database inception through March 31, 2026.

Records retrieved: n=536.

**Scopus**

**Search date:** April 5-8, 2026

**Direct graft-specific search**

TITLE-ABS-KEY((("cartilage graft" OR "cartilage grafts" OR "cartilage graft survival" OR "cartilage graft resorption" OR "cartilage transplantation" OR "cartilage implantation" OR "diced cartilage" OR "crushed cartilage" OR "morselized cartilage" OR "morselised cartilage") AND ("graft preparation" OR "cartilage preparation" OR crushing OR dicing OR morselization OR morselisation OR wrapping OR fascia OR AlloDerm OR Surgicel OR "platelet-rich plasma" OR "platelet-rich fibrin" OR "concentrated growth factor" OR "fibrin matrix" OR "autologous blood fibrin" OR perichondri* OR scaffold* OR biomaterial* OR antioxidant* OR "anti-inflammatory" OR melatonin OR "vitamin C" OR "ascorbic acid" OR glutathione OR "growth factor" OR "growth factors" OR "biologic augmentation" OR "biological augmentation"))) AND PUBYEAR < 2027 AND LIMIT-TO(LANGUAGE,"English")

**Contextual mechanistic search**

TITLE-ABS-KEY((cartilage OR chondrocyte*) AND ("oxidative stress" OR inflammation OR apoptosis OR "extracellular matrix" OR angiogenesis OR "tissue repair")) AND PUBYEAR < 2027 AND LIMIT-TO(LANGUAGE,"English")

**Limits and coverage:** English language; database inception through March 31, 2026.

Records retrieved: n=318.

**Web of Science Core Collection**

**Search date:** April 10-15, 2026

**Direct graft-specific search**

TS=((("cartilage graft" OR "cartilage grafts" OR "cartilage graft survival" OR "cartilage graft resorption" OR "cartilage transplantation" OR "cartilage implantation" OR "diced cartilage" OR "crushed cartilage" OR "morselized cartilage" OR "morselised cartilage") AND ("graft preparation" OR "cartilage preparation" OR crushing OR dicing OR morselization OR morselisation OR wrapping OR fascia OR AlloDerm OR Surgicel OR "platelet-rich plasma" OR "platelet-rich fibrin" OR "concentrated growth factor" OR "fibrin matrix" OR "autologous blood fibrin" OR perichondri* OR scaffold* OR biomaterial* OR antioxidant* OR "anti-inflammatory" OR melatonin OR "vitamin C" OR "ascorbic acid" OR glutathione OR "growth factor" OR "growth factors" OR "biologic augmentation" OR "biological augmentation")))

**Contextual mechanistic search**

TS=((cartilage OR chondrocyte*) AND ("oxidative stress" OR inflammation OR apoptosis OR "extracellular matrix" OR angiogenesis OR "tissue repair"))

**Limits and coverage:** English language; database inception through March 31, 2026.

Records retrieved: n=406.

**Google Scholar**

**Search date:** April 17-May 1, 2026

**Direct graft-specific search**

1. "cartilage graft" "platelet-rich plasma"
2. "cartilage graft" ("platelet-rich fibrin" OR "concentrated growth factor" OR "fibrin matrix")
3. ("diced cartilage" OR "crushed cartilage") (perichondrium OR scaffold OR biomaterial)
4. "cartilage graft" (antioxidant OR "anti-inflammatory" OR melatonin OR "vitamin C" OR glutathione)
5. ("cartilage graft" OR "diced cartilage") (survival OR viability OR resorption OR integration)

**Contextual mechanistic search**

Not applicable; shorter predefined combinations were used because of the less structured interface.

**Limits and coverage:** English-language records published through March 31, 2026. Results were screened in relevance order.

Records screened and exported: n=430.

Manual reference screening: Reference lists of eligible graft-specific studies and relevant reviews were screened manually. This process identified no additional unique records beyond those retrieved through database searching (n=0).

Record management: A total of 1,690 records were identified before deduplication. All records were imported into EndNote. Duplicate records were identified using EndNote's duplicate-detection function and were manually reviewed before removal; 648 duplicates were removed, leaving 1,042 records for title and abstract screening. The common publication eligibility cutoff was March 31, 2026.
