# Supplementary material for: Molecular modulation of cartilage graft survival: a systematic review and translational synthesis of local and systemic strategies
Source: JPRAS Open. 2026 Aug 10;52:128–37. doi: 10.1016/j.jpra.2026.08.001 (PMC13560031; doi:10.1016/j.jpra.2026.08.001)
Supplement: Supplementary file 2 [file mmc2.docx]

**Supplementary Table S2. SYRCLE risk-of-bias assessment**

**S2A. Selection and performance bias domains**

| **Study** | **Sequence generation** | **Baseline characteristics** | **Allocation concealment** | **Random housing** | **Caregiver/investigator blinding** |
| --- | --- | --- | --- | --- | --- |
| Cakmak et al., 2005 (3) | U | L | U | U | U |
| Kim et al., 2011 (4) | U | L | U | U | U |
| Fatemi et al., 2012 (5) | U | U | U | U | U |
| Firat et al., 2011 (6) | U | U | U | U | U |
| Kayabasoglu et al., 2015 (7) | U | L | U | U | U |
| Kemaloglu et al., 2014 (8) | U | L | U | U | U |
| Brenner et al., 2006 (10) | U | U | U | U | U |
| Manafi et al., 2012 (11) | H | L | H | U | H |
| Goral et al., 2016 (12) | U | L | U | U | U |
| Topkara et al., 2016 (13) | U | L | U | U | U |
| Aksan et al., 2021 (14) | U | L | U | U | U |
| Kulaksiz et al., 2024 (15) | U | U | U | U | U |
| Shi et al., 2020 (16) | U | L | U | U | U |
| Hapsari et al., 2022 (17) | U | L | U | U | U |

**S2B. Detection, attrition, reporting, and other bias domains**

| **Study** | **Random outcome assessment** | **Outcome-assessor blinding** | **Incomplete outcome data** | **Selective reporting** | **Other bias** |
| --- | --- | --- | --- | --- | --- |
| Cakmak et al., 2005 (3) | U | U | H | U | H |
| Kim et al., 2011 (4) | U | U | L | U | U |
| Fatemi et al., 2012 (5) | U | U | U | U | U |
| Firat et al., 2011 (6) | U | U | U | U | U |
| Kayabasoglu et al., 2015 (7) | L | U | L | U | H |
| Kemaloglu et al., 2014 (8) | U | U | L | U | U |
| Brenner et al., 2006 (10) | U | U | U | U | U |
| Manafi et al., 2012 (11) | U | U | H | U | H |
| Goral et al., 2016 (12) | U | U | L | U | U |
| Topkara et al., 2016 (13) | U | U | L | U | U |
| Aksan et al., 2021 (14) | U | U | U | U | U |
| Kulaksiz et al., 2024 (15) | U | U | U | U | U |
| Shi et al., 2020 (16) | U | U | L | U | H |
| Hapsari et al., 2022 (17) | U | U | U | U | U |

**S2C. Supporting comments**

| **Study** | **Basis for judgment** |
| --- | --- |
| Cakmak et al., 2005 (3) | Four of 33 rabbits died and were excluded. Randomization and blinding were not reported. Multiple grafts were nested within animals. |
| Kim et al., 2011 (4) | Within-animal comparison; all six rabbits were reported. Most allocation and blinding safeguards were not described. |
| Fatemi et al., 2012 (5) | Methodological reporting was insufficient for definitive judgments across most domains. |
| Firat et al., 2011 (6) | Randomization, concealment, housing, blinding, attrition handling, and prespecified outcomes were insufficiently reported. |
| Kayabasoglu et al., 2015 (7) | Randomized stereological sampling was described. Within-animal dependence was not clearly addressed. |
| Kemaloglu et al., 2014 (8) | Paired within-animal comparison; all animals were reported. Most design safeguards were not described. |
| Brenner et al., 2006 (10) | Allocation, blinding, donor matching, attrition, and clustering were insufficiently reported. |
| Manafi et al., 2012 (11) | Fixed side or position assignment made concealment and investigator blinding unlikely. Three of 15 rabbits died. |
| Goral et al., 2016 (12) | Within-animal comparison; all nine rabbits were reported. Most randomization and blinding details were unclear. |
| Topkara et al., 2016 (13) | All ten rabbits were reported. Random allocation, concealment, housing, and blinding were not clearly described. |
| Aksan et al., 2021 (14) | Within-animal comparison; other design safeguards and repeated-measures handling were insufficiently reported. |
| Kulaksiz et al., 2024 (15) | The report did not provide sufficient information for definitive judgments across the SYRCLE domains. |
| Shi et al., 2020 (16) | No attrition was reported. Multiple grafts and time points were analyzed without clear adjustment for within-animal dependence. |
| Hapsari et al., 2022 (17) | The study was described as randomized, but sequence generation, concealment, housing, blinding, and attrition handling were unclear. |

L = low risk; H = high risk; U = unclear risk. No overall numerical quality score was calculated.
