# Supplementary material for: Molecular modulation of cartilage graft survival: a systematic review and translational synthesis of local and systemic strategies
Source: JPRAS Open. 2026 Aug 10;52:128–37. doi: 10.1016/j.jpra.2026.08.001 (PMC13560031; doi:10.1016/j.jpra.2026.08.001)
Supplement: Supplementary file 3 [file mmc3.docx]

**Supplementary Table S3. Study-level descriptive outcome-direction map**

| **Study** | **Preservation/resorption** | **Oxidative stress/inflammation** | **Viability/apoptosis** | **ECM/histology** | **Integration/angiogenesis** | **Structural/biomechanical** |
| --- | --- | --- | --- | --- | --- | --- |
| Cakmak et al., 2005 (3) | M | NA | M | M | NA | M |
| Kim et al., 2011 (4) | M | NA | M | F | NA | NA |
| Fatemi et al., 2012 (5) | F | NA | NA | F | NA | F |
| Firat et al., 2011 (6) | M | NA | F | M | NA | NA |
| Kayabasoglu et al., 2015 (7) | NA | NA | F | F | NA | NA |
| Kemaloglu et al., 2014 (8) | N | NA | F | F | NA | NA |
| Brenner et al., 2006 (10) | M | NA | F | F | NA | NA |
| Manafi et al., 2012 (11) | M | F | NA | F | F | NA |
| Goral et al., 2016 (12) | NA | N | F | M | NA | NA |
| Topkara et al., 2016 (13) | NA | NA | F | F | NA | NA |
| Aksan et al., 2021 (14) | N | NA | F | N | NA | NA |
| Kulaksiz et al., 2024 (15) | NA | F | M | F | NA | NA |
| Shi et al., 2020 (16) | NA | NA | F | F | NA | F |
| Hapsari et al., 2022 (17) | NA | NA | F | F | NA | NA |

F = favorable; M = mixed; N = neutral; U = unfavorable; NA = not assessed or insufficiently reported; ECM = extracellular matrix. Each study contributes once per outcome domain. Ratings describe study-level direction only and do not represent pooled effects, effect magnitude, or statistical independence across domains.
