# Supplementary material for: Molecular modulation of cartilage graft survival: a systematic review and translational synthesis of local and systemic strategies
Source: JPRAS Open. 2026 Aug 10;52:128–37. doi: 10.1016/j.jpra.2026.08.001 (PMC13560031; doi:10.1016/j.jpra.2026.08.001)
Supplement: Supplementary file 4 [file mmc4.docx]

**Supplementary Table S4. Primary reasons for exclusion of full-text reports after eligibility assessment (n = 47)**

| **Primary reason for exclusion** | **No. of reports (n)** | **Definition** |
| --- | --- | --- |
| Wrong experimental model or outcome | 17 | Reports that did not use a relevant structurally implanted cartilage graft model and did not provide an interpretable predefined graft-related or mechanistically relevant outcome. |
| In vitro-only design | 9 | Cell-culture, tissue-culture, or laboratory studies without an in vivo implanted cartilage graft model. |
| Composite, vascularized, or complex construct without a separable cartilage graft endpoint | 12 | Studies of composite or vascularized grafts, osteochondral constructs, or tissue-engineered implants in which cartilage graft survival could not be evaluated separately. |
| No relevant technical or biologic strategy | 6 | Studies involving cartilage grafts that did not evaluate a technical graft-preparation/interface strategy or a local, systemic, or combined biologically active intervention relevant to the review question. |
| Insufficient methodological or outcome information | 3 | Reports lacking sufficient methodological detail or extractable graft-related outcome information for evidence classification and narrative synthesis. |
| **Total** | **47** | **—** |

**Abbreviation: n, number of reports.** Each excluded report was assigned to one primary exclusion category to avoid double counting. Categories were applied according to the predefined eligibility criteria described in the Methods.
